# Mechanochemical synthesis of thioureas, ureas and guanidines

**DOI:** 10.3762/bjoc.13.178

**Published:** 2017-09-01

**Authors:** Vjekoslav Štrukil

**Affiliations:** 1 Division of Organic Chemistry and Biochemistry, Ruđer Bošković Institute, Bijenička cesta 54, 10000 Zagreb, Croatia

**Keywords:** guanidines, mechanochemistry, solid state synthesis, thioureas, ureas

## Abstract

In this review, the recent progress in the synthesis of ureas, thioureas and guanidines by solid-state mechanochemical ball milling is highlighted. While the literature is abundant on their preparation in conventional solution environment, it was not until the advent of solvent-free manual grinding using a mortar and pestle and automated ball milling that new synthetic opportunities have opened. The mechanochemical approach not only has enabled the quantitative synthesis of (thio)ureas and guanidines without using bulk solvents and the generation of byproducts, but it has also been established as a means to develop "click-type" chemistry for these classes of compounds and the concept of small molecule desymmetrization. Moreover, mechanochemistry has been demonstrated as an effective tool in reaction discovery, with emphasis on the reactivity differences in solution and in the solid state. These three classes of organic compounds share some structural features which are reflected in their physical and chemical properties, important for application as organocatalysts and sensors. On the other hand, the specific and unique nature of each of these functionalities render (thio)ureas and guanidines as the key constituents of pharmaceuticals and other biologically active compounds.

## Introduction

**Scheme 1:**
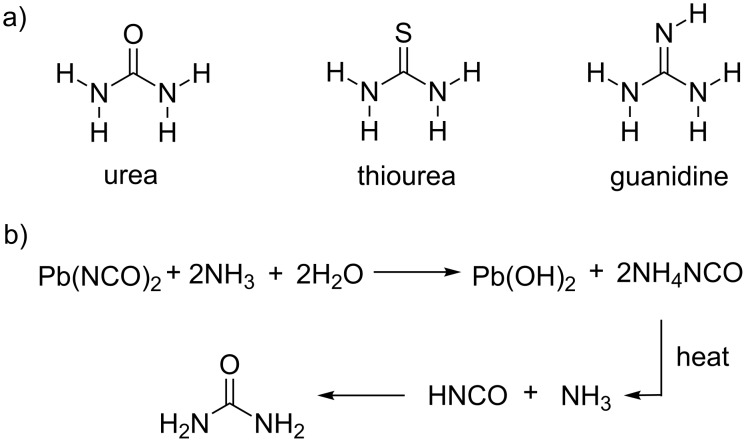
a) Schematic representations of unsubstituted urea, thiourea and guanidine. b) Wöhler's synthesis of urea.

The urea molecule played the central role in the development of organic chemistry since its first documented synthesis in 1828 when the German chemist Friedrich Wöhler prepared it starting from ammonium cyanate (Scheme 1) [1]. This simple, yet intriguing transformation of an inorganic chemical into an organic product, at that time only available from living organisms, was in contradiction with the prevailing doctrine of vitalism, which was in the years to come abandoned enabling a rapid evolution of organic chemistry in the 19th century.

**Figure 1:**
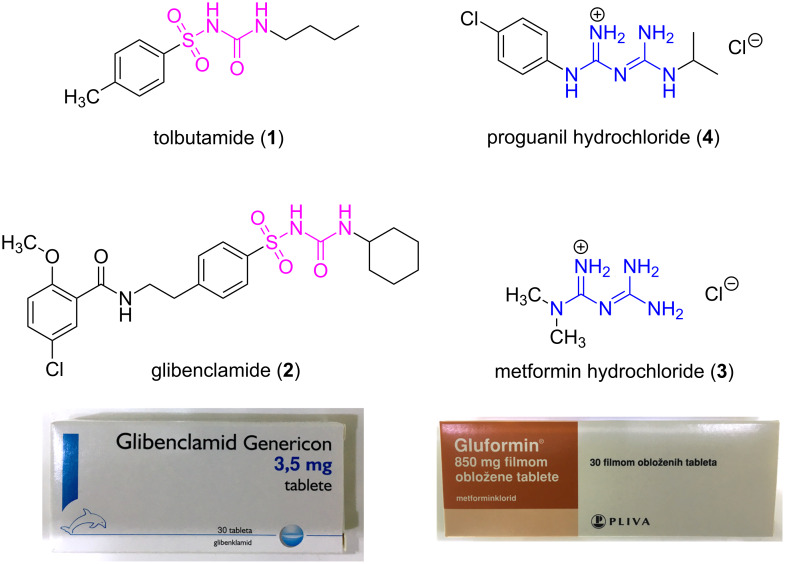
Antidiabetic (**1–3**) and antimalarial (**4**) drugs derived from ureas and guanidines currently available in the market.

During the 20th century, synthetic routes to (thio)ureas and guanidines and their properties were extensively investigated, especially in terms of biological activity [2–5]. Most notable examples of pharmaceutically relevant ureas and guanidines available on the market are shown in Figure 1. The antidiabetic drugs tolbutamide (**1**) and glibenclamide (**2**), which belong to the class of sulfonylureas, and guanidine-derived metformin (**3**) are among the top selling oral hypoglycemics globally. Proguanil (**4**), a biguanide derivative, is widely prescribed to treat malaria, a disease that took over 430 000 lives in 2015 [6].

**Scheme 2:**
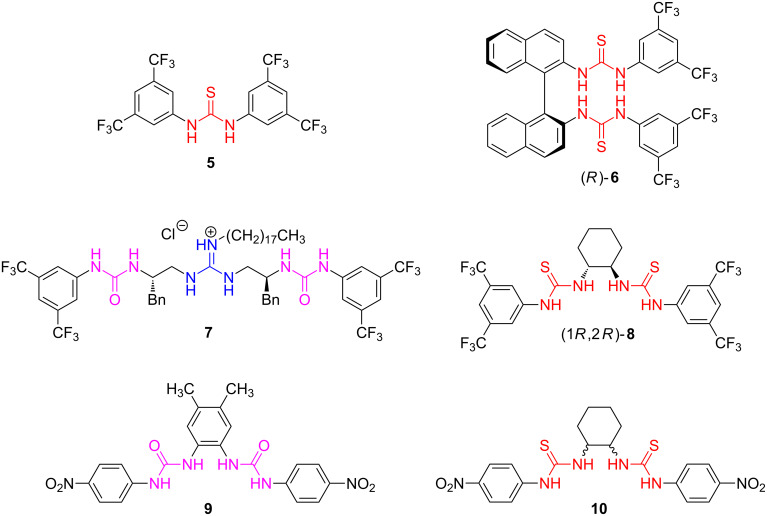
The structures of some representative (thio)urea and guanidine organocatalysts **5**–**8** and anion sensors **9** and **10**.

In the past 20 years, molecules with incorporated (thio)urea and guanidine subunits, due to their ability to coordinate other molecules and ions via N–H hydrogen bonding, have also been considered as organocatalysts and anion sensors [7–12]. In Scheme 2, several examples of (thio)urea- and guanidine-based organocatalysts are shown.

Green Chemistry, which aims at turning chemical reactions into more effective and sustainable processes with high conversions of the starting materials and no byproduct formation, has emerged as a mainstream paradigm in chemical research in the past 25 years. Anastas and Warner have proposed 12 Principles of Green Chemistry as a guide to help making chemical processes more environmentally friendly [13–14]. Many of the requirements contained in these principles (e.g., prevention, atom economy, energy efficiency, catalysis, safe synthesis) can be met if the reactions are transferred from the solution into the solid state. In a typical solid state organic synthesis, reactants are simply ground together in a mortar using a pestle, where the mechanical force is exerted by a hand (manual grinding) [15]. Whereas mechanochemistry [16], at least on the laboratory scale, is usually associated with mortar and pestle processing, this approach suffers from several issues, such as non-constant energy input leading to inhomogeneous mixing and transfer of mechanical energy, irreproducibility, exposure to air/humidity (unless the experiment is carried out in a glovebox) and finally the compromised safety for the researcher. These drawbacks can be eliminated or substantially reduced by the application of automated ball mills. The precise control of parameters such as reaction time, milling frequency, number and size of milling balls, type of milling media (stainless steel, zirconia, teflon, plastic) and even milling atmosphere allows reproducible solid state syntheses in such instruments. The progress made over the past 15 years has transformed grinding or milling from a purely physical tool for mechanical processing into a synthetic method of choice when one wishes to conduct chemical reactions in an environmentally-friendly fashion [17–18]. In this respect, there have been several turning points in the development of solid-state mechanochemistry. The first key discovery was made by Jones et al. who discovered the rate-accelerating effect of adding small catalytic quantities of a liquid phase to a mixture treated by manual grinding or ball milling [19]. What was in the beginning termed as "solvent-drop grinding" (SDG) eventually became "liquid-assisted grinding" or LAG, now a well-established method for improving the outcome of mechanochemical reactions [20]. In continuation of this research, Friščić et al. introduced the so called "ion and liquid-assisted grinding" or ILAG by recognizing the effect of cations such as Na^+^, K^+^ or NH_4_^+^ or anions like Cl^−^, NO_3_^−^ and SO_4_^2−^ on the formation of polymorphs during LAG synthesis of metal-organic frameworks [21]. Recently, Jones et al. employed polymeric macromolecular catalysts, e.g., PEG 200 and PEG 10000 as solid auxiliaries to enhance crystallization under LAG mechanochemical conditions in "polymer and liquid-assisted grinding" or POLAG [22–23]. While the focus in these investigations has been on the improvement of the macroscopic parameters such as the reaction yield, another aspect of mechanochemical reactions that is becoming important for further development in the field is the mechanism of solid-state reactions. To be able to see beyond the usual ex situ analyses of mechanochemical reactions, modifications of the milling equipment had to be made. Since these are solid-state reactions, powder X-ray diffraction (PXRD) using synchrotron radiation was suitable as the analytical tool to monitor the changes during ball milling on a microscopic level in real time [24]. In this way, the first in situ observations of mechanochemical reactions were performed which has led to the discovery of reactive intermediates, new phases and novel topologies in systems previously studied only by ex situ analyses [25–26]. To overcome the inability of PXRD to provide structural information on amorphous materials, a method based on real time in situ Raman spectroscopy was devised [27]. Finally, these two in situ techniques have been successfully merged to allow simultaneous monitoring of mechanochemical reactions by PXRD and Raman spectroscopy [28–29].

## Review

### Mechanochemical synthesis of (thio)ureas

#### Thioureas

**Scheme 3:**
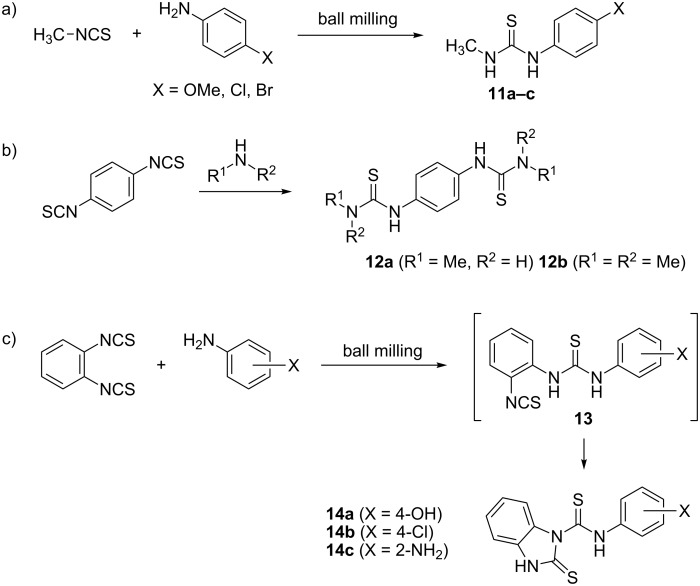
Solid-state reactivity of isothiocyanates reported by Kaupp [30].

In a paper by Kaupp et al. a study on the reactivity of gaseous and solid amines with solid isothiocyanates was described [30]. The authors carried out gas–solid reactions via vapour digestion and solid–solid reactions by means of ball milling. To ensure that the investigated reactions were genuine solid-state processes, in some cases the milling was performed at low temperatures (−30 °C) using an in-house ball mill equipped with a cooling jacket. As isothiocyanate component, liquid phenyl isothiocyanate and solid methyl, 1-naphthyl, 4-bromophenyl and 4-nitrophenyl isothiocyanates were screened. While ammonia, methylamine and dimethylamine were selected as gaseous amines and quantitatively afforded thioureas at pressures of 0.4–1 bar and reaction temperatures of −30 °C to rt , solid anilines such as 4-methoxy, 4-chloro and 4-bromoaniline were reacted in the solid-state under ball milling conditions at rt. In all three cases the authors reported 100% yields (Scheme 3a).

Starting from solid phenylene-1,4-diisothiocyanate and methylamine or dimethylamine, bis-thioureas **12a** and **12b** were quantitatively prepared by gas–solid reactions. When phenylene-1,2-diisothiocyanate was used in solid-state reactions with 4-hydroxyaniline, 4-chloroaniline and 1,2-phenylenediamine, benzimidazolidine-2-thiones **14a**–**c** were isolated in 100% yields via cyclization of an unstable intermediate **13** (Scheme 3b,c). Compared to the solvent-free synthesis, the corresponding solution reactions resulted in lower yields (81–95%). Li and co-workers conducted a mortar-and-pestle synthesis of 14 diarylthioureas by reacting 4-ethoxy-, 4-chloro- and 4-bromophenyl isothiocyanates with several anilines. After manual grinding for 5–40 min, the crude products were recrystallized from ethanol or acetone, and dried under vacuum to afford the thioureas in 89–98% yield [31].

**Scheme 4:**
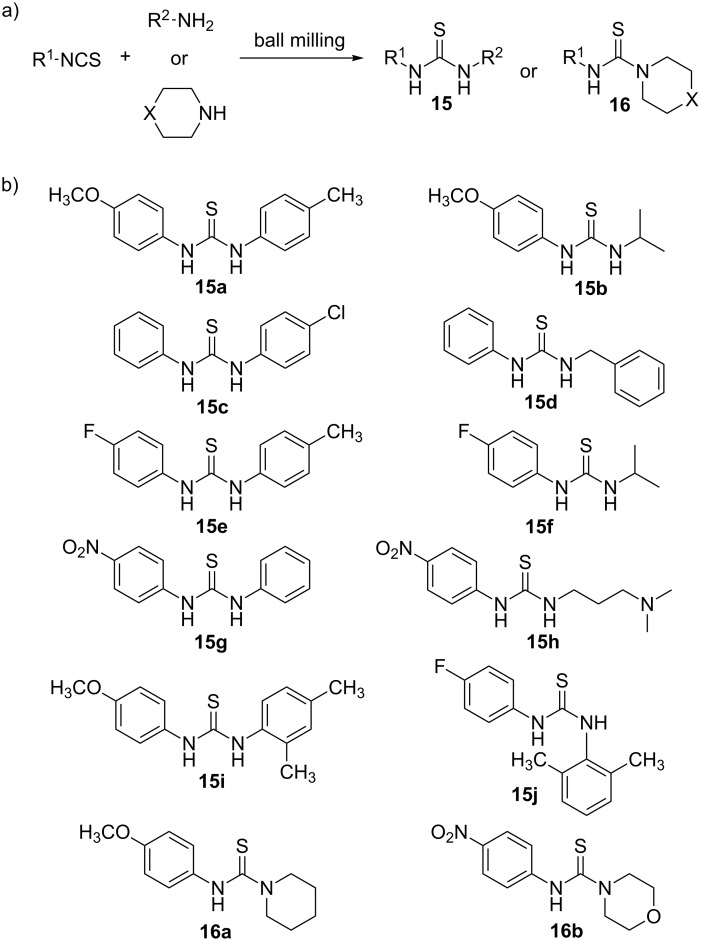
a) Mechanochemical synthesis of aromatic and aliphatic di- and trisubstituted thioureas by click-coupling of amines with aromatic isothiocyanates. b) Selected examples of thioureas synthesized in quantitative yields.

Inspired by these findings, our group decided to explore the reactivity pattern of aromatic and aliphatic amines and aromatic isothiocyanates during mechanochemical synthesis of 49 symmetrical and non-symmetrical *N*,*N'*-disubstituted thioureas [32]. For this purpose, a range of amines and isothiocyanates were screened with electron-donating and electron-withdrawing groups attached to aromatic rings. The reactions were performed in a 1:1 stoichiometry by manual grinding in a mortar and by automated ball milling in a laboratory mixer mill. Also, the performance of solvent-free or neat grinding was compared to liquid-assisted grinding, as well as the effect of the physical state of the reactants (liquid or solid) on the isolated yields. In general, manual grinding for 5–45 min (typically 15–20 min to ensure quantitative conversion) worked well with ≥99% yields in all cases regardless of the electronic effects exerted by different substituents, or liquid or solid character of the starting materials. Interestingly, in most cases a simple manual mechanical agitation of the reaction mixtures in a mortar provided products after only a few minutes of grinding. However, the combination of an electron-withdrawing group in the amine (lower nucleophilicity) and an electron-donating group in the isothiocyanate component (lower electrophilicity) led to prolonged grinding times necessary to achieve quantitative conversion. The reaction time in these cases was successfully reduced by LAG, providing *N*,*N'*-disubstituted thioureas in quantitative yields. In contrast to mortar-and-pestle synthesis, automated ball milling at 30 Hz using a single 12 mm stainless steel ball afforded the desired products quantitatively in 10 minutes, demonstrating its efficiency for a rapid and general synthesis of thioureas via click-type amine–isothiocyanate coupling reaction (Scheme 4).

In the case of secondary amines (piperidine, morpholine and thiomorpholine) and sterically hindered amines (2,4- and 2,6-dimethylanilines), ball milling again resulted in ≥99% yields in 10 minutes, except for the reactions involving 4-methoxyphenyl isothiocyanate, which required 45 minutes of manual grinding and 15 or 45 minutes of milling, due to its diminished electrophilicity.

In the context of these results, it is reasonable to assume that the solvent-free microwave synthesis of diarylthioureas described by Li et al. actually proceeded in the solid-state before having been exposed to microwave irradiation for 1.5–4.5 minutes. In their paper, the authors state: "Aryl isothiocyanate (1 mmol) and aromatic primary amine (1 mmol) were mixed thoroughly in an agate mortar" [33]. Considering the established reactivity pattern of electron-withdrawing aryl isothiocyanates with anilines used for the synthesis of *N*,*N'*-disubstituted thioureas, thorough mixing in an agate mortar typically leads to the formation of the products in a couple of minutes.

**Figure 2:**
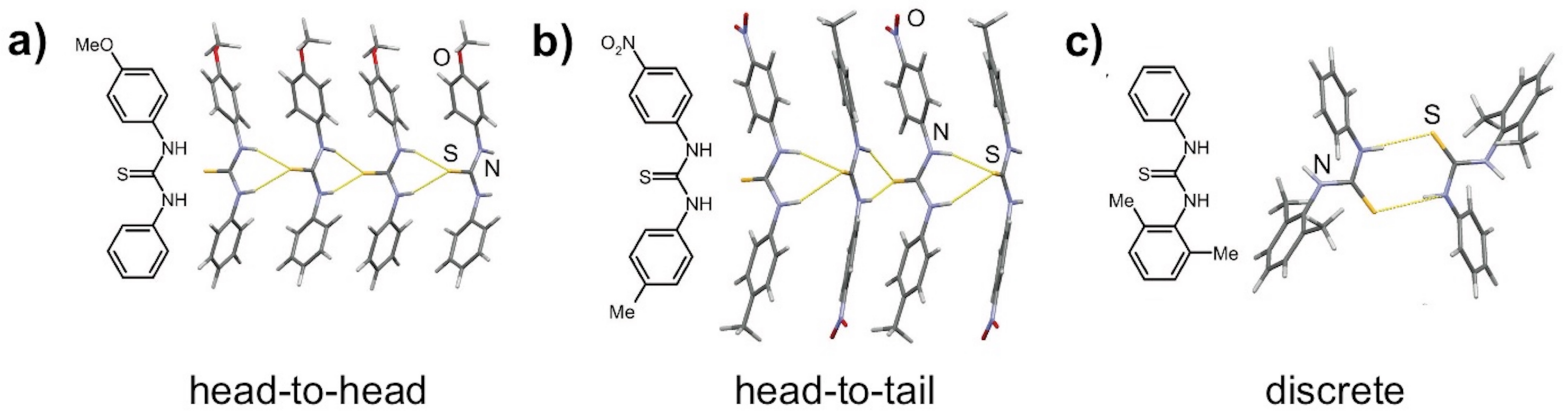
The supramolecular level of organization of thioureas in the solid-state.

As an extension of the mechanochemical click-coupling of amines with isothiocyanates, the thiourea products were structurally characterized by solid-state analytical methods such as powder X-ray diffraction (PXRD) and solid-state NMR (ssNMR) spectroscopy. In this way, mechanochemical organic synthesis and solid-state analysis are incorporated into the paradigm of solvent-free synthetic organic research laboratory, where all the steps from synthesis to structural characterization are carried out without using bulk solvents. The systematic PXRD analyses of 49 thioureas revealed that thioureas, on a supramolecular level, organize into three types of self-assembly motifs based on N–H···S hydrogen bonds: corrugated chains of head-to-head or head-to-tail aligned molecules and discrete centrosymmetric dimers based on the R_2_^2^(8) supramolecular synthon in the case of sterically hindered thioureas (Figure 2).

The crystal structures of *N*,*N'*-diarylthioureas linked in chains via N–H···S hydrogen bonds can further be subdivided into two structural families. The chains in the family I are stacked in a parallel fashion with a width of the supramolecular stack corresponding to the Bragg diffraction angle range 5–7° and the (200) reflection, intensity of which is a result of diffraction from the sulfur atoms in neighbouring stacks.

**Figure 3:**
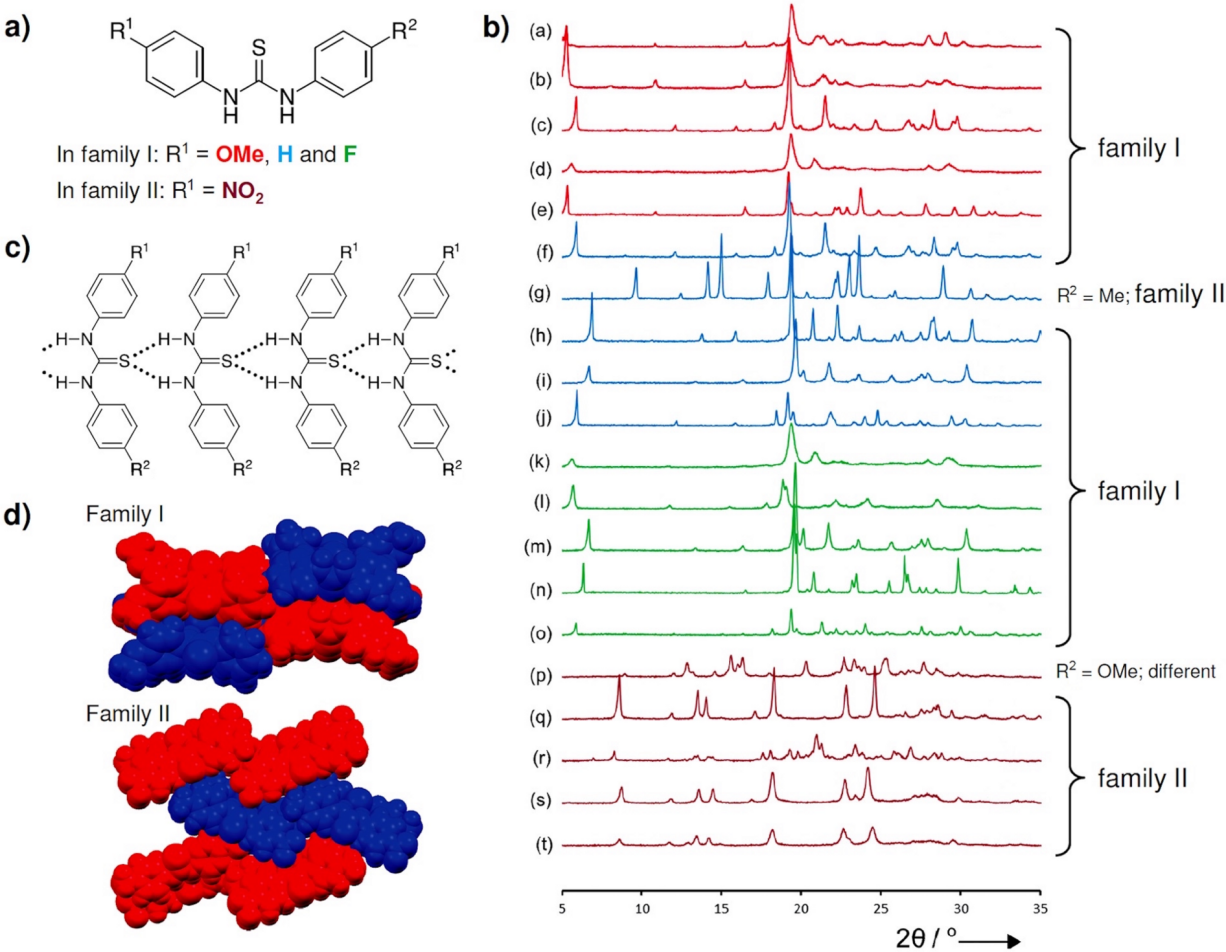
The supramolecular level of organization of thioureas in the solid-state.

In the structural family II, the characteristic (110) reflection is slightly shifted and appears at the Bragg diffraction angle range 8–10°. The infinite hydrogen-bonded chains are arranged in a herringbone pattern with an angle of 44° between neighbouring stacks (Figure 3).

**Scheme 5:**
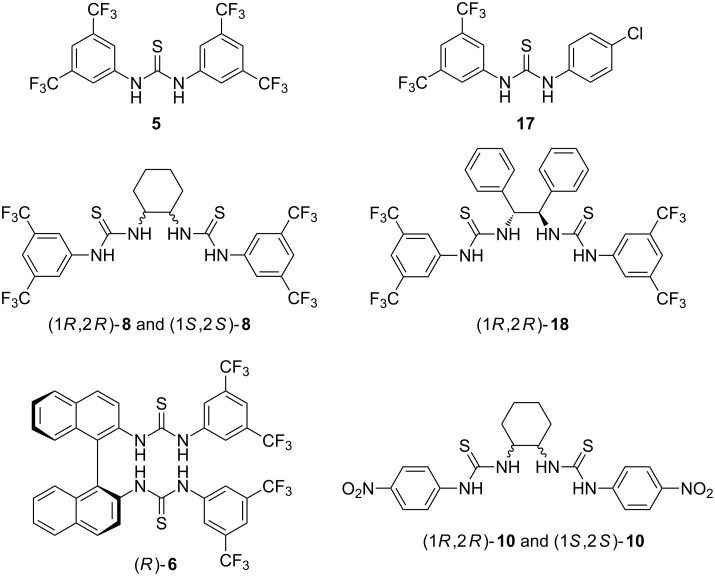
Thiourea-based organocatalysts and anion sensors obtained by click-mechanochemical synthesis.

In the follow-up paper, the ball milling approach was then applied for a quantitative click-mechanosynthesis of thiourea-based organocatalysts and anion sensors (Scheme 5) [34]. The demonstrated efficiency of mechanochemical milling synthesis of thioureas was exploited for a quantitative transformation of enantiomerically-pure chiral reagents, availability of which in a laboratory is dictated by their high costs. For that reason, we looked into the possibility to convert these reagents into functional chiral molecules with the highest synthetic efficiency. The privileged 3,5-di(trifluoromethyl)phenyl motif in organocatalyst design was first introduced by reacting 3,5-di(trifluoromethyl)phenyl isothiocyanate with 3,5-di(trifluoromethyl)aniline and 4-chloroaniline in a 1:1 ratio under LAG conditions using methanol as the grinding liquid. This led to quantitative formation of the Schreiner's catalyst **5** and thiourea **17** as evidenced by the disappearance of the characteristic –N=C=S stretching band between 2000 and 2200 cm^–1^ in the FTIR-ATR spectra.

The isothiocyanate was then coupled with other chiral diamines such as enantiomers of *trans*-1,2-diaminocyclohexane, (1*R*,2*R*)-(+)-1,2-diphenylethylenediamine and (*R*)-(+)-1,1′-binaphthyl-2,2′-diamine in a stoichiometric ratio. The corresponding chiral bis-thiourea organocatalysts were isolated in ≥99% yields after only 20 minutes (60 min in the case of binaphthylthiourea) of neat grinding or LAG. Interestingly, while the solution synthesis of (1*R*,2*R*)-**8** in THF followed by recrystallization from a hexane/ethyl acetate mixture gave previously unrecognized but highly stable 1:1 ethyl acetate solvate, the mechanochemical synthesis led to the pure non-solvated catalyst. The mechanochemically prepared achiral thiourea **5** as well as enantiomers (1*R*,2*R*)-**8** and (1*S*,2*S*)-**8** were next screened as catalysts in Morita–Baylis–Hillman reaction, and their performance matched the previously published catalytic activity. An analogous click-type reaction between 4-nitrophenyl isothiocyanate and *trans*-1,2-diaminocyclohexane quantitatively afforded enantiomeric (1*R*,2*R*)-**10** and (1*S*,2*S*)-**10** bis-thioureas which were tested as cyanide anion sensors in DMSO solution.

Our group continued the research on the solid-state synthesis of thioureas focusing now on the reactivity of sterically hindered *ortho*-phenylenediamine (*o*-pda) with isothiocyanates [35]. Whereas Kaupp's approach to prepare a bis-thiourea derivative by milling 1,2-diisothiocyanate with two equivalents of an amine failed and resulted in the formation of benzimidazolidine-2-thiones **14a**–**c** by cyclization of the mono-thiourea intermediate **13** (Scheme 3), our reaction design was based on the click-coupling of *o*-pda with either one or two equivalents of phenyl, 4-methoxyphenyl, 4-chlorophenyl or 4-nitrophenyl isothiocyanate.

**Scheme 6:**
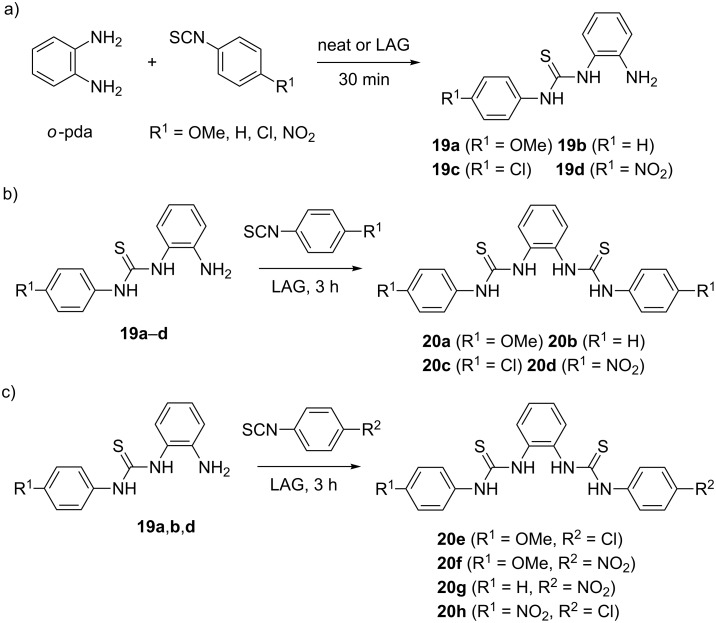
Mechanochemical desymmetrization of *ortho*-phenylenediamine.

**Scheme 7:**
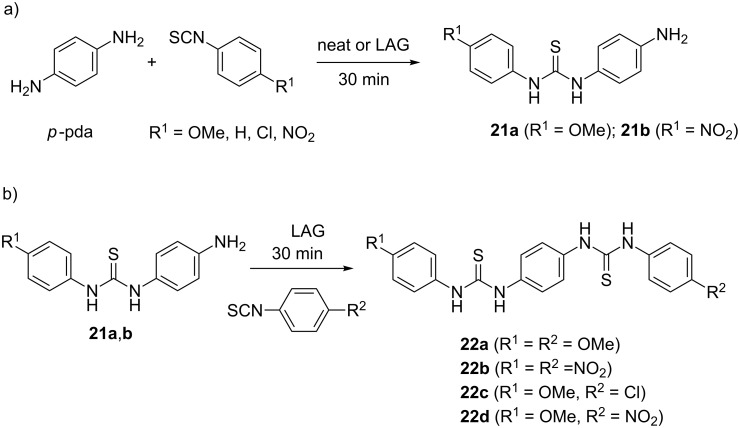
Mechanochemical desymmetrization of *para*-phenylenediamine.

In the 1:1 reaction, solvent-free mechanosynthesis selectively provided stable mono-thioureas **19a**–**d** in ≥95% after 30 minutes (Scheme 6a). When the reactants were milled in a 1:2 ratio for 3 hours (9 hours for 4-methoxy derivative), the symmetrical bis-thioureas **20a**–**d** were isolated in excellent ≥95% yields (Scheme 6b). Such a selective transformation of *o*-pda into mono-thioureas enabled the synthesis of non-symmetrical bis-thioureas **20e**–**h** by a one-pot two-step mechanochemical reaction, without the need to isolate and purify the mono-thiourea intermediates. For example, the reaction of 4-methoxy **19a**, phenyl **19b** and 4-nitro mono-thiourea **19d**, with the second equivalent of an isothiocyanate furnished the non-symmetrical products in ≥99% after 3 hours of LAG using methanol (Scheme 6c). In the case of *para*-phenylenediamine (*p*-pda) where steric hindrance is absent, the desymmetrization was more challenging. It was only achieved in 97% **21a** in the reaction with less reactive 4-methoxyphenyl isothiocyanate under NaCl dilution and LAG using ethyl acetate. When highly reactive 4-nitrophenyl isothiocyanate was utilized, a mixture of mono- **21b** and bis-thioureas **22b** was isolated (Scheme 7).

However, the corresponding 1:2 reactions quantitatively gave symmetrical bis-thioureas **22a** and **22b** after only 30 minutes of LAG. Also, the non-symmetrical thioureas **22c** and **22d** were prepared by coupling mono-thiourea **21a** with 4-chloro- and 4-nitrophenyl isothiocyanates. This study demonstrated that solid-state ball milling can efficiently be employed for desymmetrization of *ortho*- and *para*-phenylenediamines, enabling selective functionalization of small symmetrical molecules through the extension of molecular structure in a one-pot two-step mechanochemical sequence.

**Scheme 8:**
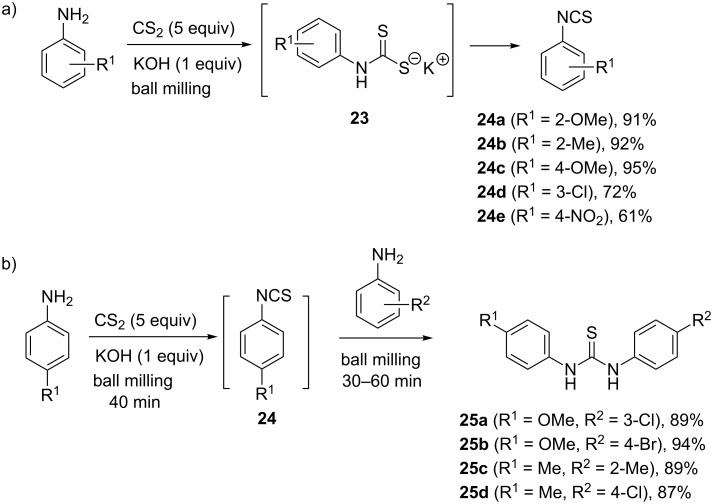
a) Selected examples of a mechanochemical synthesis of aromatic isothiocyanates from anilines. b) One-pot two-step synthesis of some non-symmetrical thioureas **25a**–**d**.

Another typical synthetic method for the preparation of thioureas, particularly if the desired isothiocyanate is not available, is the condensation of an amine with carbon disulfide [36]. This reaction proceeds through the formation of a dithiocarbamate salt in the first step, which can be isolated or desulfurized in situ to provide the isothiocyanate reagent. Without isolation, the isothiocyanate undergoes a reaction with the amine and produces the thiourea product. Such an approach for thiourea synthesis under mechanochemical ball milling conditions was investigated by Zhang et al. [37]. In their procedure, anilines were mechanochemically transformed into isothiocyanates **24** in the presence of 5.0 equivalents of CS_2_ or symmetrical thioureas (in the presence of 1.0 equiv CS_2_) by potassium hydroxide-promoted decomposition of the intermediate dithiocarbamate salt **23** (Scheme 8a). In comparison with 24 h reactions carried out in solvents (CH_2_Cl_2_, THF, acetone, methanol, DMF, DMSO or neat CS_2_), the mechanochemical synthesis was rapid and furnished electron-rich isothiocyanates in high yields in 40–45 minutes (e.g., **24a**–**c**). On the other hand, anilines bearing electron-withdrawing substituents were less reactive, resulting in prolonged milling (90 minutes) and only moderate yields of the isothiocyanate products **24d**,**e**.

The observation that isothiocyanates were major products when excess CS_2_ (5.0 equiv) was employed, while the stoichiometric reaction with 1.0 equiv of CS_2_ switched the reactivity and afforded symmetrical thioureas in good to excellent yields, prompted the authors to conduct a two-step synthesis of non-symmetrical thioureas **25** (Scheme 8b). In the first step, electron-rich 4-methoxyaniline or 4-methylaniline were ball milled with CS_2_ (5.0 equiv) for 40 minutes, followed by the click-coupling reaction of the second equivalent of an aniline with the intermediate isothiocyanate. In this way, non-symmetrical thioureas **25a**–**d** were synthesized and isolated in high 87–94% yields.

**Scheme 9:**
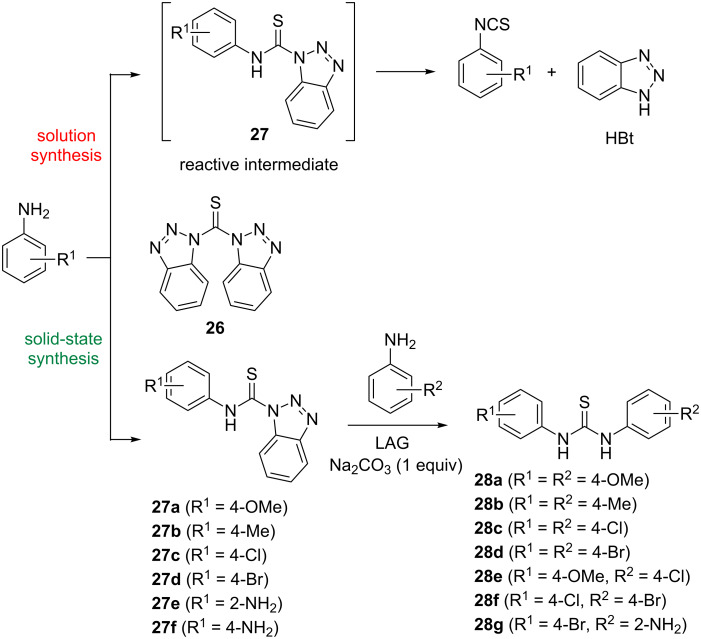
In solution, aromatic *N*-thiocarbamoyl benzotriazoles **27** are unstable and decompose to isothiocyanates and benzotriazole (HBt). Mechanochemical solvent-free synthesis yields **27a**–**f** as bench-stable solids, that are readily converted to thioureas **28a**–**g**.

Instead of using thiophosgene and CS_2_ as corrosive and hazardous liquid reactants that require special handling, solid thioacylating reagents such as 1,1'-thiocarbonyldiimidazole and bis(1-benzotriazolyl)methanethione (**26**) are air-stable and easier to work with during thiourea synthesis. While their solution chemistry in thioacylation and thiocarbamoylation reactions has been documented [38–40], the reactivity of these compounds in the solid-state mechanochemical transformations remained unexplored. Our attention was also caught by the fact that thiocarbamoylation in solution using **26**, provided only alkyl derivatives in 60–98% yield. For aromatic derivatives **27**, it has been explicitly stated in the literature that these compounds are very reactive intermediates and immediately decompose to isothiocyanates and 1*H*-benzotriazole (HBt). With this in mind, we investigated the possibility to run the thiocarbamoylation reaction of *para*-substituted anilines as nucleophilic aromatic substrates with bis(1-benzotriazolyl)methanethione (**26**) under ball-milling conditions (Scheme 9) [41]. The application of in situ Raman spectroscopy monitoring of mechanochemical reactions, in combination with solid-state characterization through FTIR-ATR, PXRD and ssNMR analyses, confirmed that mechanochemistry afforded the elusive aromatic *N*-thiocarbamoyl benzotriazoles **27** in quantitative yields after only 10 minutes of LAG and a simple aqueous work-up.

Furthermore, conducting the reaction in two steps, where the thiocarbamoyl benzotriazole was prepared in the first step followed by the addition of the second equivalent of aniline, led to non-symmetrical thioureas **28e**–**g** in ≥97% yields (Scheme 9).

**Scheme 10:**
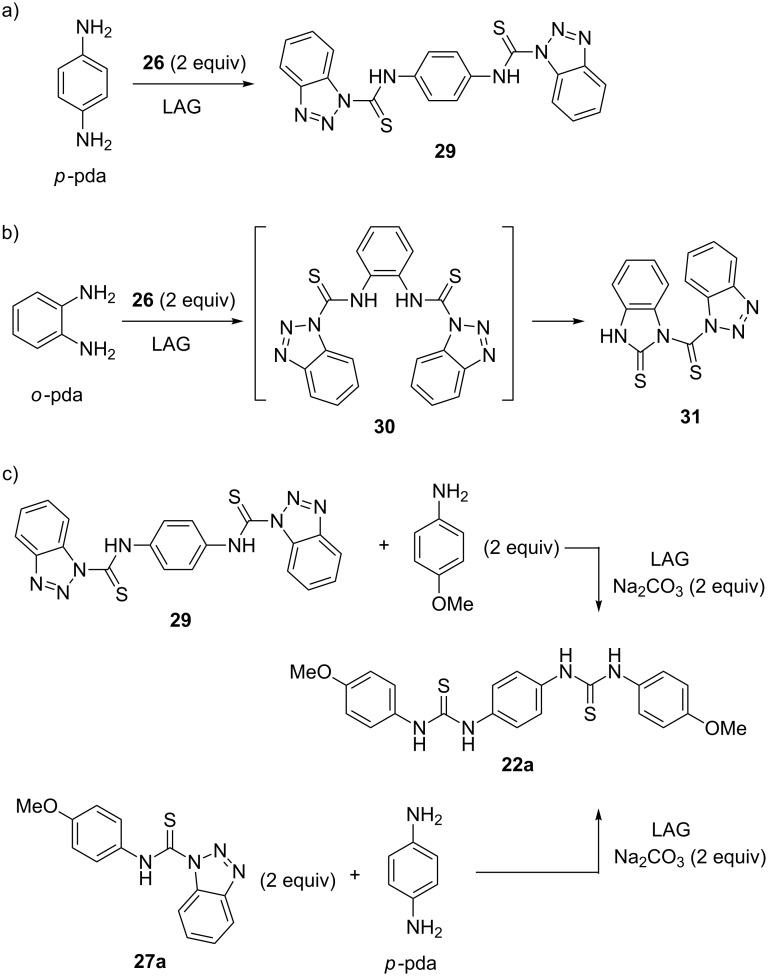
Mechanosynthesis of a) bis-thiocarbamoyl benzotriazole **29** and b) benzimidazole thione **31**. c) Synthesis of bis-thiourea **22a** from mono- (**27a**) and bis- (**29**) *N*-thiocarbamoyl benzotriazoles.

Treating *p*-pda with two equivalents of **26** gave 99% of bis-thiocarbamoyl benzotriazole **29**, a masked 1,4-phenylene diisothiocyanate equivalent. In contrast, the analogous reaction of *o*-pda failed to give the desired *ortho*-bis-thiocarbamoyl benzotriazole **30** after 2 hours of LAG. The isolated product was identified as benzimidazole thione **31**, formed presumably by an intramolecular cyclization of the unstable bis-derivative **30** (Scheme 10a and b).

**Figure 4:**
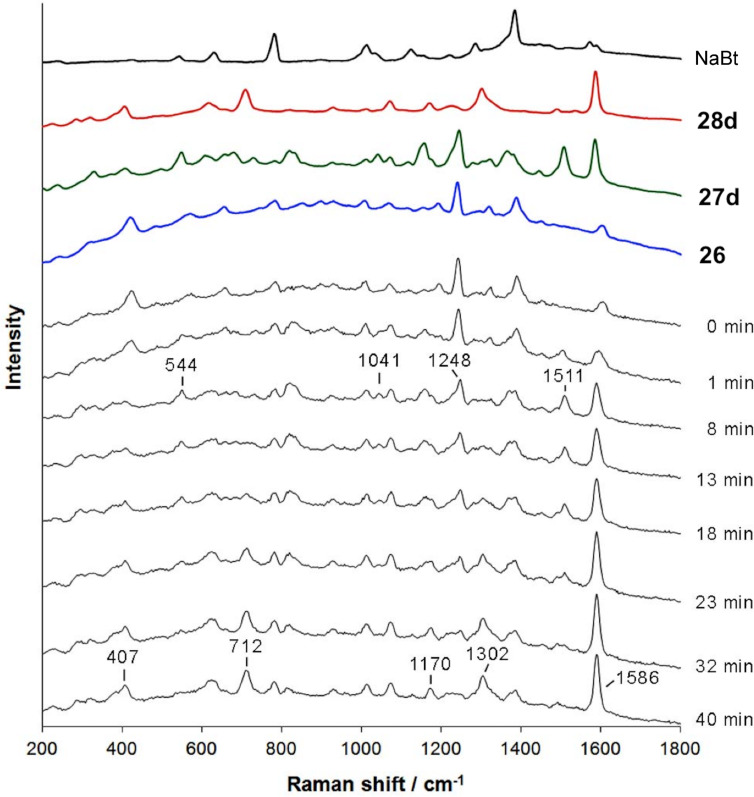
In situ Raman spectroscopy monitoring the synthesis of thiourea **28d** in the solid-state. *N*-Thiocarbamoyl benzotriazole **27d** was identified as the intermediate, with the characteristic bands at 544, 1041, 1248, and 1511 cm^−1^ appearing ca. 2 min into milling and disappearing with the formation of **28d**.

Since *N*-thiocarbamoyl benzotriazoles can be regarded as synthetic equivalents of isothiocyanate reagents, they were utilized for the solid-state synthesis of thioureas by milling **26** with two equivalents of aniline in the presence of sodium carbonate as the base. After 10 minutes, symmetrical aromatic thioureas **28a**–**d** were obtained in almost quantitative yields. The in situ Raman monitoring of a 1:2 mixture of **26** and 4-bromoaniline, which results in the formation of symmetrical bis(4-bromophenyl)thiourea **28d** revealed thiocarbamoyl benzotriazole **27d** as the reactive intermediate (Figure 4).

Starting from **27a** or **29**, bis-thiourea **22a** can be quantitatively accessed by controlling the aniline to thiocarbamoyl benzotriazole stoichiometry (Scheme 10c).

Apart from providing another example of stoichiometry-controlled synthesis under mechanochemical conditions, these results have also demonstrated the power of solid-state milling as a synthetic tool that enables the synthesis and isolation of molecular species as bench-stable chemicals, that are normally considered as reactive intermediates in solution environment.

**Scheme 11:**
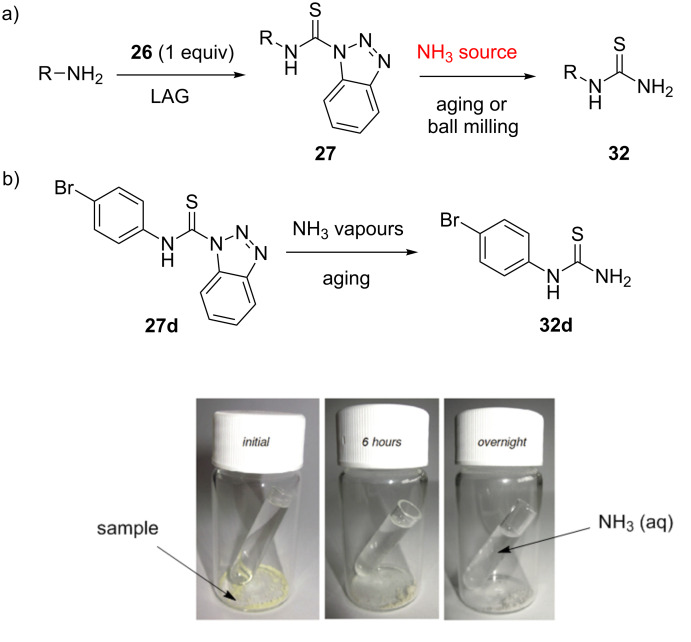
a) The proposed synthesis of monosubstituted thioureas **32**. b) Conversion of *N*-thiocarbamoyl benzotriazole **27d** to thiourea **32d** by aging in ammonia vapours.

The observed reactivity of thiocarbamoyl benzotriazoles prompted us to examine their reaction with ammonia, as a potential route to primary monosubstituted thioureas **32** [42]. Primary thioureas are typically prepared in solution from benzoyl chloride and ammonium thiocyanate or by condensation of amine hydrochlorides and potassium thiocyanate [43–44]. Our strategy was to synthesize the desired thiocarbamoyl benzotriazole in the first step, and then carry out the amination reaction in the second step using the appropriate ammonia source (Scheme 11a). As a test reaction, the amination of 1-[(4-bromophenyl)thiocarbamoyl]benzotriazole (**27d**) in ammonia vapours by the so called aging or vapour digestion was selected. It was evident by the colour change of the sample that the chemical reaction occured which was also confirmed by FTIR-ATR analysis (Scheme 11b). The decrease of band intensities of thiocarbamoyl benzotriazole **27d** at 1588, 1520, 1157, 1143, 968, 924 and 494 cm^−1^ was accompanied by the appearance of characteristic absorption bands of *N*-(4-bromophenyl)thiourea (**32d**) at 1617 and 509 cm^−1^. Several other thiocarbamoyl benzotriazoles were also quantitatively transformed to primary thioureas by this method.

**Scheme 12:**
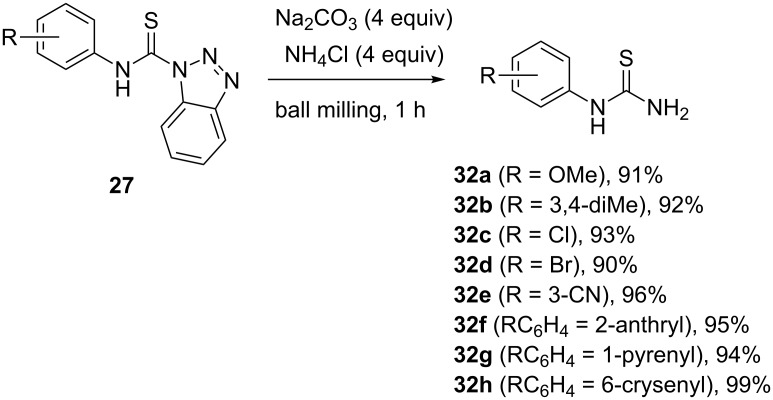
A few examples of mechanochemical amination of thiocarbamoyl benzotriazoles by in situ generated ammonia.

For the purpose of performing the amination reaction in a ball mill, ammonia gas was generated in situ by milling the thiocarbamoyl substrate with a mixture of sodium carbonate and ammonium chloride. This mixture released ammonia gas during milling and allowed the amination reaction to take place under solvent-free mechanochemical conditions. Following a simple aqueous work-up and filtration, the desired primary thioureas **32** were isolated in quantitative yields. The amination reaction was then performed on a number of substrates, ranging from simple mono- and disubstituted anilines, benzylamines and polyaromatic amines such as anthracene-, phenanthrene-, pyrene- and crysenamine (Scheme 12).

An interesting feature of LAG synthesis of monosubstituted thioureas was that water as the grinding liquid, or aqueous solutions of organic solvents where *x*(H_2_O) > 0.8, significantly affected the conversion of thiocarbamoyl benzotriazole **27d**. In the case of LAG with water, the quantitative IR analysis revealed only 3% conversion to thiourea **32d**, whereas LAG with aqueous ammonia solution as a source of NH_3_ (instead of Na_2_CO_3_/NH_4_Cl mixture) gave a poor yield of 24%. The phenomenon of LAG inhibition was explained by strong hydrogen-bonding solvation of NH_3_ molecules in water which are likely to form cluster species NH_4_^+^(H_2_O)*_n_*, not reactive in the amination reaction.

#### Ureas

**Scheme 13:**
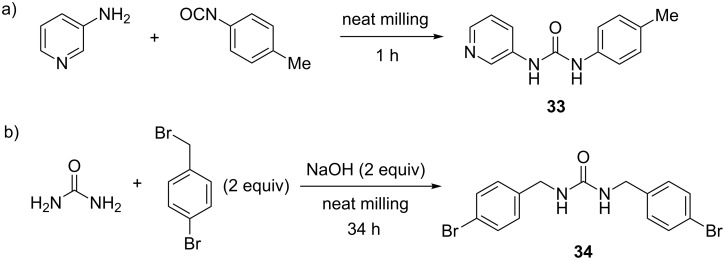
Mechanochemical synthesis of a) anion binding urea **33** by amine-isocyanate coupling and b) dialkylurea **34** by alkylation of unsubstituted urea.

Just as thioureas are typically synthesized by coupling reaction between amines and isothiocyanates, ureas as oxygen analogues are prepared from the corresponding isocyanates. This approach was employed in the synthesis of anion binding 1-(pyridin-3-yl)-3-*p*-tolylurea (**33**) reported by Swinburne and Steed in 2009 [45]. This compound was found to bind anions individually and as part of a tripodal anion receptor. In contrast to solution synthesis in dichloromethane for 12 hours, the mechanochemical solvent-free coupling of 3-aminopyridine and 4-methylphenyl isocyanate provided the target urea sensor after milling for 60 minutes at 18 Hz (Scheme 13a). Monitoring the progress of the reaction by ex situ ^1^H NMR spectroscopy in DMSO-*d_6_* revealed that the reaction reached completion after only 30 minutes of ball milling with a conversion greater than 90%. Although the purity of the sample was satisfactory enough to be further used as-synthesized, an analytically pure sample could easily be obtained by simple washing with CH_2_Cl_2_. The mechanochemically prepared urea **33** was next used in the synthesis of tri- and tetrapodal anion receptors, again by exploiting the solid-state LAG ball milling approach.

Mack et al. looked into the formation of a dialkylurea from the parent urea in the context of the mechanochemical formation of dialkyl carbonates from metal carbonates [46]. Whereas urea is normally considered as unreactive compound, the authors succeeded to activate it under ball-milling conditions by using two equivalents of sodium hydroxide. Deprotonation of the N–H group increased the nucleophilicity of the nitrogen atoms, enabling the nucleophilic displacement reaction with two equivalents of 4-bromobenzyl bromide to yield di(4-bromobenzyl)urea **34** in 41%, after a total of 34 hours of milling (Scheme 13b). This transformation showed that ball milling could potentially be applied to increase the nucleophilicity of an otherwise poorly reactive compound.

**Scheme 14:**
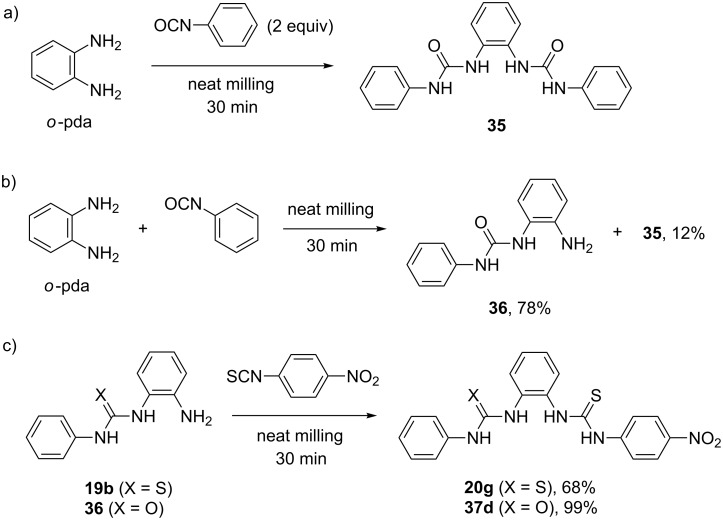
a) Solvent-free milling synthesis of the bis-urea anion sensor **35**. b) Non-selective desymmetrization of *o*-pda with phenyl isocyanate. c) Different reactivity of mono-thiourea **19b** and mono-urea **36** under mechanochemical conditions.

In the course of our studies on mechanochemical desymmetrization, we also investigated the reaction of *o*-pda and mono-urea **36** with phenyl isocyanate under the milling conditions used for the synthesis of bis-thioureas [35]. A known bis-urea anion sensor **35** was prepared in quantitative yield in 30 minutes by milling *o*-pda with phenyl isocyanate in a 1:2 molar ratio. However, in the 1:1 reaction, a mixture of mono-urea **36** (78%), bisurea **35** (12%) and *o*-pda (10%) was isolated, thus contrasting the reactions involving isothiocyanates (Scheme 14a,b). On the other hand, milling mono-urea **36** with one equivalent of *p*-nitrophenyl isothiocyanate for 30 minutes quantitatively yielded the mixed urea–thiourea **37d**. When mono-thiourea **19b** was used under these conditions, the conversion to bis-thiourea **20g** was 68% due to lower reactivity of mono-thioureas in comparison with mono-ureas (Scheme 14c).

**Scheme 15:**
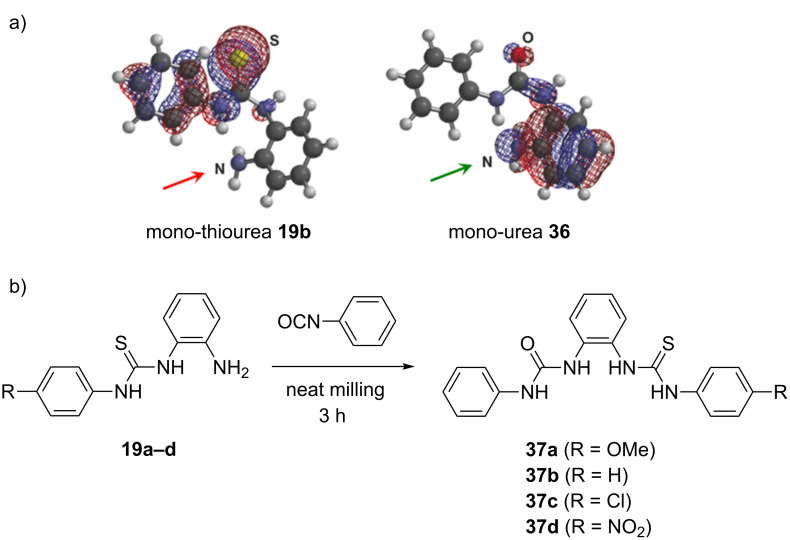
a) HOMO−1 contours of mono-thiourea **19b** and mono-urea **36**. b) Mechanochemical synthesis of hybrid urea-thioureas **37a–d**.

Quantum mechanical frontier molecular orbital (FMO) analysis of mono-(thio)ureas allowed us to rationalize different reactivity patterns observed experimentally. The FMO analysis of **19b** showed more electron density on the sulfur atom compared to the nitrogen of the amino group in the highest occupied molecular orbital (HOMO−1). In contrast, the coefficient was larger on the NH_2_ nitrogen atom in HOMO−1 of mono-urea **36** thus making it more nucleophilic in the addition reaction to isocyanates (Scheme 15a). The ability to selectively convert *o*-pda into non-symmetrical mono-thioureas provided an opportunity to synthesize hybrid urea–thiourea derivatives **37a**–**d** in a one-pot, two-step mechanochemical solvent-free process. After ball milling for three hours, the addition of phenyl isocyanate (1 equiv) to mono-thioureas **19a**–**d** quantitatively yielded the mixed urea–thioureas **37a**–**d** (Scheme 15b), which could also be prepared by a ‘‘reverse’’ mechanosynthesis starting from the mono-urea **36**.

**Scheme 16:**
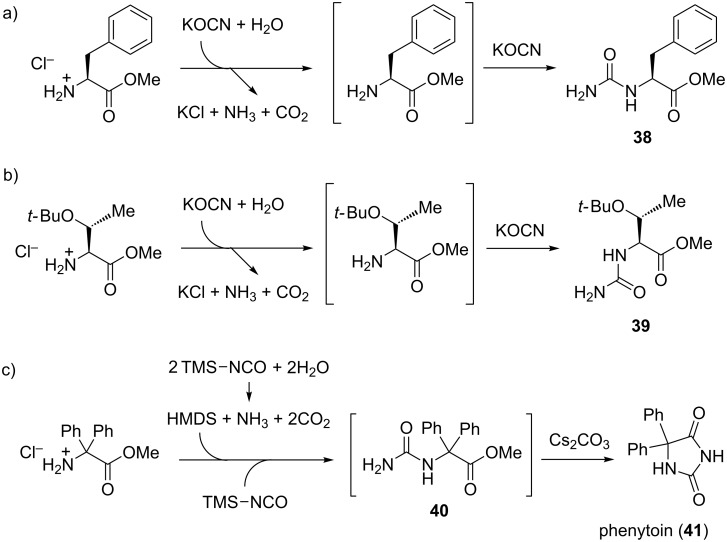
Synthesis of ureido derivatives **38** and **39** from KOCN and hydrochloride salts of a) L-phenylalanine methyl ester and b) L-threonine(O*t*-Bu) methyl ester. c) Mechanochemical synthesis of the anti-epileptic drug phenytoin (**41**).

An interesting approach, published by Colacino et al., to introducing urea functionality in amino acid methyl esters by mechanochemically reacting them with potassium cyanate (KOCN) was described [47]. The ureido products arising from this reaction are intermediates in what is known in the literature as the Urech synthesis of 1,3-unsubstituted hydantoins. The in situ basic conditions, necessary for the deprotonation of the amino acid methyl ester hydrochloride salts in order to make the amino group nucleophilic, were generated by the hydrolysis of KOCN. Following the addition reaction with KOCN starting from hydrochloride salts of L-phenylalanine or L-(*tert*-butyl)threonine methyl esters, ureido derivatives **38** and **39** were isolated in high yields (96 and 97%, respectively; Scheme 16a,b). A number of other α-amino methyl esters, quaternary amino methyl esters or β-amino methyl esters were also successfully converted to intermediate ureas (without isolation) and cyclized in the presence of a base to 5-substituted hydantoins in good to excellent yields.

Then the ball milling methodology was applied to the synthesis of phenytoin (**41**), a known antiepileptic drug. In this case, KOCN had to be replaced with trimethylsilyl isocyanate (TMS-NCO) which generated the strong hexamethyldisilazane (HMDS) base upon hydrolysis. Deprotonation of sterically hindered diphenylglycine methyl ester hydrochloride followed by the hydrolysis of the TMS group provided the ureido-intermediate **40** after 8 hours of milling at 450 rpm. The cyclization of **40** with Cs_2_CO_3_ for 3 hours finally afforded phenytoin in an excellent 84% isolated yield (Scheme 16c).

The introduction of a sulfonyl group on the urea framework has been found to be the crucial structural modification in the development of the 1st generation antidiabetic drugs such as tolbutamide and chlorpropamide or the 2nd generation drugs like glibenclamide (Figure 1). These molecules were interesting synthetic targets for our mechanochemical approach which is based on a stoichiometric base-assisted or copper-catalyzed coupling of sulfonamides and iso(thio)cyanates [48].

**Scheme 17:**
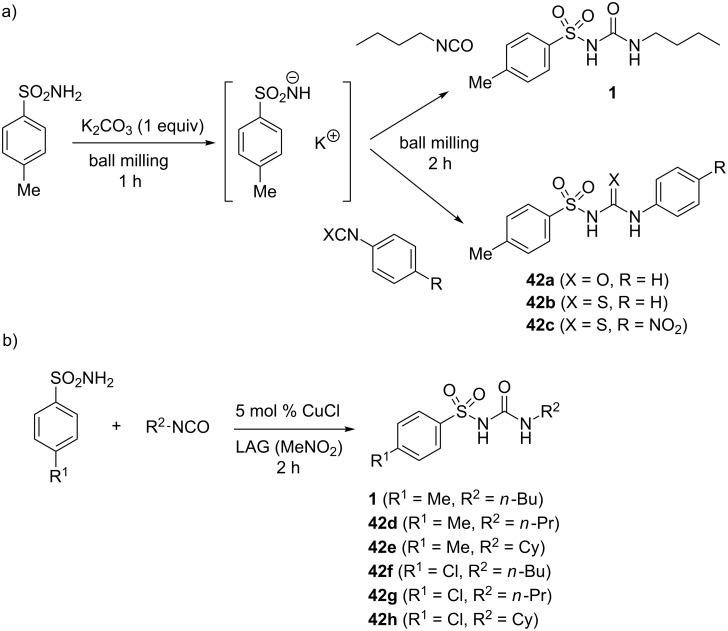
a) K_2_CO_3_-assisted synthesis of sulfonyl (thio)ureas. b) CuCl-catalyzed solid-state synthesis of sulfonyl ureas.

For that purpose, 0.5–1 equiv of potassium carbonate as the base was necessary to deprotonate the sulfonamide and thus increase its reactivity. After milling for 2 hours with the corresponding iso(thio)cyanate, the sulfonyl (thio)ureas **42a**–**c** were isolated in excellent yields, for example the drug tolbutamide (**1**) in 92% (Scheme 17a). Sulfonylureas could also be obtained by coupling of sulfonyl isocyanates with amines which was demonstrated by an efficient solvent-, base- and catalyst-free synthesis of tolbutamide (93%) starting from *p*-toluenesulfonyl isocyanate and *n*-butylamine. However, this approach was not further pursued due to the air-sensitivity and corrosive nature of the sulfonyl isocyanate reagent. In addition, these reagents are generally unavailable in comparison with sulfonamides, many of which are air-stable commercial chemicals [49].

In order to avoid using stoichiometric quantities of a base, a mechanochemical catalytic approach to tolbutamide with CuCl as the catalyst was explored (Scheme 17b). Two hours of neat grinding of an equimolar mixture of *p*-toluenesulfonamide and *n*-butyl isocyanate in the presence of 5 mol % of CuCl resulted in 68% of the desired product **1**. Increasing the catalyst loading to 20 mol % improved the yield to 91%. Conducting the ball milling under LAG conditions enabled the CuCl loading to be kept as low as 5 mol %. Using nitromethane as the most effective grinding liquid, tolbutamide (**1**) was isolated in 90% yield. The optimization study also revealed that other sources of copper such as Cu(II) salts and Cu(0) in the powder form catalyzed the reaction. Most notably, the reaction proceeded in an excellent 87% yield even without external copper catalyst, only by using a brass milling ball. The catalyst was removed from the crude reaction mixture by briefly milling it with aqueous sodium ethylenediaminetetraacetate.

**Scheme 18:**
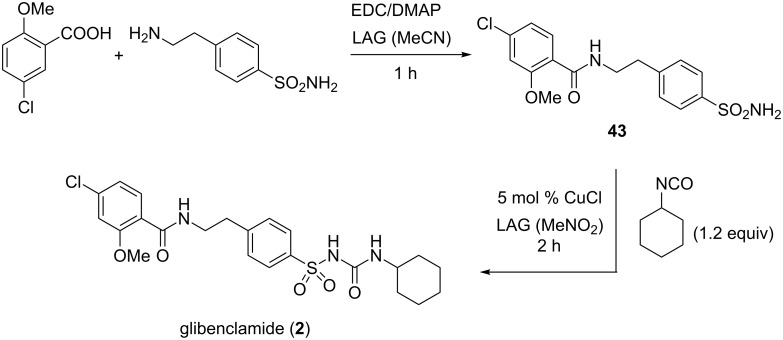
Two-step mechanochemical synthesis of the antidiabetic drug glibenclamide (**2**).

Glibenclamide (**2**) as our next target was more complex as it also posesses the additional amide functionality. We envisaged a two-step mechanochemical synthesis of glibenclamide, where in the first step the amide bond would be constructed by amine–carboxylic acid coupling, followed by catalytic sulfonamide–isocyanate coupling. The mechanochemical EDC-mediated amide bond formation [50] was successful and provided the intermediate **43** in 74% yield. In the second step, coupling of the sulfonamide intermediate **43** with 1.2 equivalents of cyclohexyl isocyanate in the presence of 5 mol % of CuCl and nitromethane as the grinding liquid in LAG (η = 0.25 μL mg^−1^), quantitatively yielded glibenclamide (**2**, Scheme 18).

**Scheme 19:**
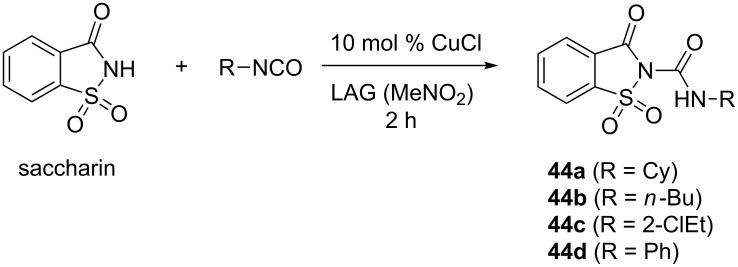
Derivatization of saccharin by mechanochemical CuCl-catalyzed addition of isocyanates.

The same group reported on the use of the famous artificial sweetener saccharin in the mechanochemical coupling with cyclohexyl, *n*-butyl, 2-chloroethyl and phenyl isocyanates [51]. The corresponding saccharyl ureas **44a**–**d** were isolated in high yields after CuCl-catalyzed (10 mol %) LAG for 2 hours (Scheme 19). These several examples of sulfonylureas nicely demonstrate that ball milling is also a very powerful environmentally-friendly synthetic tool in medicinal chemistry.

### Mechanochemical synthesis of guanidines

#### Guanidines

**Scheme 20:**
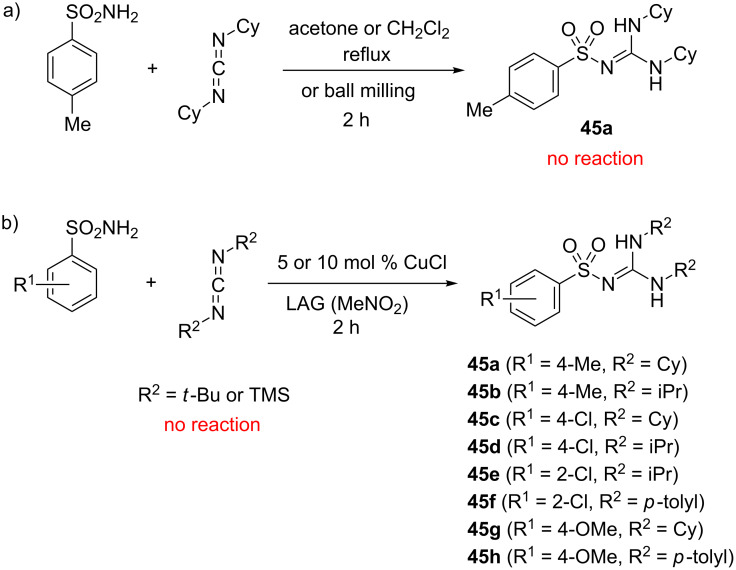
a) Unsuccessful coupling of *p*-toluenesulfonamide and DCC in solution and by neat/LAG ball milling. b) CuCl-catalyzed synthesis of some sulfonyl guanidines under LAG milling conditions.

The success of mechanochemical synthesis of sulfonylureas by the coupling of sulfonamides with isocyanates led us to investigate the reactivity of sulfonamides with carbodiimides as another example of the heterocumulene system [52]. The attempted addition of *p*-toluenesulfonamide to *N*,*N'*-dicyclohexylcarbodiimdie (DCC) failed in solution, but also under solvent-free and LAG mechanochemical conditions (Scheme 20a). However, when this mixture was milled for 2 hours neat in the presence of 5 mol % of CuCl, the product **45a** was obtained in 81%, while LAG (nitromethane, η = 0.25 μL mg^−1^) resulted in almost quantitative yield. Interestingly, the catalysis in solution did not work, hence representing the first example of carbon–nitrogen coupling reaction that was accessible only by mechanochemistry. This discovery suggests that milling not only enhances the previously known reactivity, but it also has the potential for reaction discovery and development.

Applying the standard milling conditions, a series of sulfonylguanidines was synthesised in ≥90% yields from alkyl or aromatic carbodiimides and aromatic sulfonamides (Scheme 20b). Sterically hindered carbodiimides such as *tert*-butyl and trimethylsilyl derivatives displayed no reactivity. With 2-naphthyl and *p*-nitrophenylsulfonamides as poorly reactive compounds, additional LAG screening experiments were required to establish the optimal reaction conditions by switching to acetone as the grinding liquid, prolonging the milling time to 4 hours and increasing the catalyst loading to 10–20 mol %. In general, there was no reactivity without CuCl, in solution or in the presence of a base instead of CuCl, implying that CuCl activated the carbodiimide component during this catalytic reaction.

**Scheme 21:**
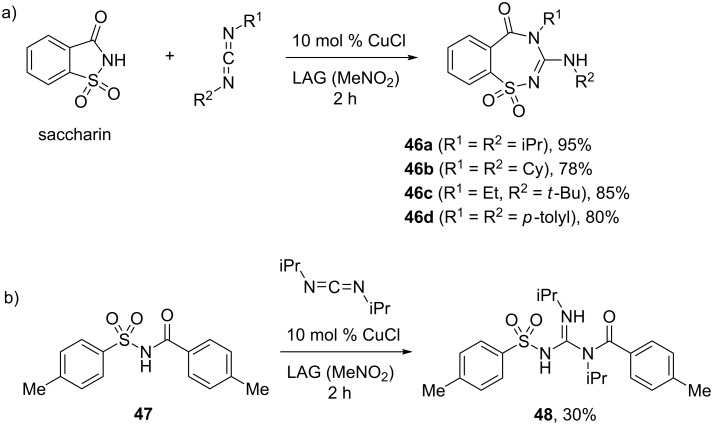
a) Expansion of the saccharin ring by mechanochemical insertion of carbodiimides. b) Insertion of DIC into the linear analogue **47**.

Tan and Friščić further developed this mechanochemical synthetic strategy and applied it to a previously unknown carbodiimide insertion into sulfonimides, resulting in two-atom ring expansion and chain extension reactions [51]. Saccharin was selected as a model cyclic sulfonimide substrate, while 4-methyl-*N*-tosylbenzamide was employed as an acyclic analogue. Single crystal X-ray diffraction analyses of the products obtained by firstly reacting saccharin with several carbodiimides in solution (ethyl acetate, acetone or acetonitrile) revealed the formation of the 7-membered benzo[1,2,4]thiadiazepine ring in all cases. For example, the product **46b** (Scheme 21), which was previously characterized as a simple guanidine adduct between saccharin and DCC, arose from the DCC insertion into the 5-membered saccharin ring.

Under mechanochemical conditions, solvent-free or LAG milling of saccharin with *N*,*N'*-diisopropylcarbodiimide (DIC) failed to afford the desired product. However, the addition of 10 mol % of CuCl catalyst led to the quantitative formation of benzo[1,2,4]thiadiazepine **46a** after 2 hours, as evidenced by FTIR-ATR and PXRD analyses of the crude reaction mixture. Other carbodiimides also smoothly underwent the mechanochemical insertion, e.g., DCC (78%), *N*-ethyl-*N'*-*tert*-butylcarbodiimide (85%) and di-*p*-tolylcarbodiimide (80%, Scheme 21a). The performance of the reaction was not affected even on >1 g scale. Milling 4-methyl-*N*-tosylbenzamide (**47**) with DIC and CuCl (10 mol %) for 2 hours resulted in the insertion of the carbodiimide into the C–N bond of benzamide and the formation of *N*-acylsulfonylguanidine **48** extended by two atoms (Scheme 21b).

#### Biguanides

**Scheme 22:**
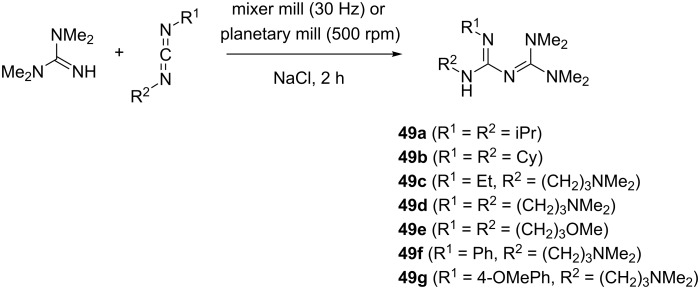
Synthesis of highly basic biguanides by ball milling.

The attachment of an amidine subunit onto the guanidine core, which is typically accomplished by the addition of a carbodiimide molecule, leads to a biguanide framework. In a paper by Margetić and Eckert-Maksić, several non-classical preparative methods were evaluated for the synthesis of highly basic hexasubstituted biguanides **49a**–**g** (Scheme 22) [53]. One of the techniques employed was mechanochemical ball milling in a mixer mill and a planetary mill. In the case of the mixer mill, the reaction conditions were 2 hours at 30 Hz frequency using a 12 mm stainless steel ball, while in the planetary mill 50 × 3 mm balls were used at 500 rpm. Sodium chloride was added as the solid auxiliary to facilitate the mass transfer during milling. Under these conditions, 1,1,3,3-tetramethylguanidine as the nucleophile was reacted with 1.3 equiv of dialkyl- and alkylaromatic carbodiimides.

With less reactive dialkyl carbodiimides the yields were poor, however, the introduction of an aromatic substituent (phenyl or 4-methoxyphenyl) in the carbodiimide component significantly increased the reactivity resulting in >90% conversion and >80% isolated yields of biguanides **49f** and **49g** (Table 1).

**Table 1:** The efficiency of mixer and planetary ball milling in the synthesis of biguanides **49a–g**.^a^

biguanide	conversion [%]	

	mixer mill	planetary mill
	
**49a**	15	40
**49b**	–	5
**49c**	traces	–
**49d**	<5^b^	–
**49e**	44^b^	–
**49f**	95^b^ (82)	–
**49g**	94^b^ (86)	–

^a^Mixer mill: 12 mm ball, 30 Hz, 2 h; planetary mill: 50 × 3 mm balls, 500 rpm; NaCl (Na_2_SO_4_ for **49f** and **49g**) as the solid auxiliary. ^b^Milling time 1 h.

## Conclusion

Mechanochemical solid-state ball milling has enabled the efficient, high-yielding, rapid and operationally-simple syntheses of (thio)ureas and guanidines. The utility of these compounds as synthetic intermediates, organocatalysts and anion sensors, in combination with specific reactivity of iso(thio)cyanates or carbodiimides with amines as suggested by the experimental and theoretical observations, has kept the focus of mechanochemical synthesis primarily on thioureas. Still, the structural diversity of the molecules presented herein testify that mechanochemistry can be utilized to successfully cope with the challenges of modern synthetic organic chemistry, in terms of quantitative conversion of chiral substrates, desymmetrization of small molecules, metal-catalyzed reactions and molecular rearrangements. Many examples demonstrate that the mechanochemical approach to synthesis enhances the already described reactivity patterns, but also allows the development and discovery of novel reactions under milling conditions. The possibility to conduct mechanochemical reactions in near-quantitative yields has eliminated the need for excess reagents, transforming them into stoichiometric, or even catalytic processes. Finally, as an inherently solvent-free methodology, mechanochemistry has made the usage of bulk solvents obsolete in the synthesis steps, thus simplifying the isolation procedures as well. With the principal synthetic routes to (thio)ureas and guanidines in the solid-state now established, the next challenge of incorporation of these simple structural units into more complex molecular systems by mechanochemistry is expected.
